# Supplementary material for: Estimating the burden of severe malarial anaemia and access to hospital care in East Africa
Source: Nat Commun. 2023 Sep 14;14:5691. doi: 10.1038/s41467-023-41275-w (PMC10502125; doi:10.1038/s41467-023-41275-w)
Supplement: Supplementary file 1 — Supplementary information [file 41467_2023_41275_MOESM1_ESM.pdf]

Supplementary information

Model

We estimated the prevalence of malaria (as determined by microscopy test) and severe anaemia in children aged 0.5-5 years in country i, $\mathrm{MASA}_{0.5-5i}$, as

$$\mathrm{MASA}_{0.5-5i} = a_i e^{-b}, \tag{S1}$$

$$\mathrm{logit}(a_i) = \alpha + \beta_i, \tag{S2}$$

$$\log(b) = \gamma - \delta Pf\mathrm{Pr}_{2-10} \tag{S3}$$

where a_i represents the upper asymptote of prevalence in country i. The log odds of a_i is composed of the linear combination of the intercept, α, and the country-level random effect, β_i. The shape of the curve is defined by b where the global parameters, γ and δ, represent the shift (displacement with respect to $Pf\mathrm{Pr}_{2-10}$) and growth rate respectively.

We corrected for the prevalence of non-malarial severe anaemia (negative malaria test and Hb <5), $\mathrm{SA}_{0.5-5i}$, to estimate the prevalence of acute severe malarial anaemia, SMA

$$\mathrm{SMA}_{0.5-5i} = \frac{(\mathrm{MASA}_{0.5-5i} - Pf\mathrm{Pr}_{2-10}\mathrm{SA}_{0.5-5i})}{(1 - \mathrm{SA}_{0.5-5i})}, \tag{S4}$$

and age-standardise the prevalence to match the age distribution of the hospitalisation data

$$\mathrm{SMA}_{0.25-9i} = \mathrm{SMA}_{0.5-5i} \frac{(a_{11} - a_{10}) \int_{a_{00}}^{a_{01}} \mathrm{P}(x)dx}{(a_{01} - a_{00}) \int_{a_{10}}^{a_{11}} \mathrm{P}(x)dx} \tag{S5}$$

where a_{10} , a_{11} are the lower and upper input ages respectively (0.5 years and 5 years) and a_{00} , a_{01} are the lower and upper output ages respectively (0.25 years and 9 years), in months. $\mathrm{P}(x)$ is the lognormal density function capturing the age distribution of SMA cases, with parameters mean = 2.83 and sd = 0.79, on the log scale [1].

We estimated the annual incidence per 1000 children, $\mathrm{iSMA}_{0.25-9i}$, as

$$\mathrm{iSMA}_{0.25-9i} = 365 \cdot 1000 \cdot \left(\frac{\zeta \mathrm{SMA}_{0.25-9i}}{1 - \mathrm{SMA}_{0.25-9i}}\right), \quad \text{S6}$$

where ζ is the recovery rate from SMA. The hospitalised annual incidence per 1000 children in country i, hospital j is then estimated

$$\mathrm{hSMA}_{0.25-9ij} = g_{ij}\mathrm{iSMA}_{0.25-9j}\,, \quad \text{S7}$$

$$\log(g_{ij}) = \eta_i + \theta k_{ij} \quad \text{S8}$$

where g_{ij} is the odds of an SMA case accessing hospital j in country i. The $\log(g_{ij})$ is composed of the linear combination of the country level estimate of access, η_i, and a modifier of access, θ, with respect to the distance to the hospital, k_{ij}.

We jointly fitted the models of MASA prevalence and hospitalised SMA incidence in a Bayesian framework assuming that the probability of a child in country i having malaria and severe anaemia, P_i^{MASA}, is:

$$\mathrm{MASA}_{0.5-5i} \sim \mathrm{Ber}(P_i^{MASA}) \quad \text{S9}$$

$$\beta_i \sim \mathrm{Norm}(0, \sigma^2). \quad \text{S10}$$

The probability of child in country i having non-malarial severe anaemia P_i^{SA} is:

$$SA_{0.5-5i} \sim \mathrm{Ber}(P_i^{SA}) \quad \text{S11}$$

the number of children hospitalised with SMA in country i, hospital j , N_{ij}^{hSMA}, is:

$$N_{ij}^{hSMA} \sim \mathrm{NBinom}(\mathrm{hSMA}_{0.25-9ij}, \mu), \quad \text{S12}$$

parameterised via the mean and size, μ, parameters.

and the survey reported probability of hospitalisation given MASA in country i is:

$$P_i^{h|\text{MASA}} \sim \text{Ber}\left(\frac{g_i}{1+g_i}\right), \quad \text{S13}$$

where g_i is the odds of an SMA case accessing hospital in country i, assuming the average distance to hospital for country i, in the Paton *et al* data.

We account for survey structure design by weighting the likelihood calculations for P_i^{MASA} and P_i^{SA} by the DHS household survey-weights. Model fits were run with 30,000 burn in and 30,000 sampling iterations for each of 4 chains. Convergence and sampling efficiency were monitored via the Rhat statistic (all < 1.005) and effective sample sizes respectively (all >500). Uncertainty is represented using Bayesian equal tail 95% credible intervals.

Model checking and validation

Few estimates of the community, or whole population incidence of SMA have been published which could be used for model validation. One resource not reliant on hospital data is from the RTS,S vaccine trial where incidence of SMA was recorded in actively-followed community cohorts for 11 sites in 7 countries [2]. We used our fitted model to predict total SMA cases given $Pf\text{Pr}_{2-10}$ [3], and observation person-years (children aged 5-17 months) [2], to compare with the observed SMA incidence in the trial. For countries in the RTS,S trial, which were not represented in the available DHS/MIS data that our model was fitted to, we assumed that the country-level random effect, β_i, was set to the mean, 0 and the prevalence of non-malarial anaemia and hospitalisation odds were set to the mean estimates from Kenya, Tanzania, and Uganda.

Infection timescale

Data from rodent models with varying levels of immunity and parasitaemia suggest minimum Hb occurring 4-13 days post-patency, recovering quickly in the following 10-14 days [4]. Similarly, studies in monkey models show recovery in most cases within 7-14 days following parasite clearance via self-resolution or treatment [5]. Whilst not representative of children in endemic areas, monitoring of Hb levels in patients infected with *Plasmodium falciparum* to treat neurosyphilis show similar trends

with decreasing Hb in the first 6-14 days, followed by recovery of erythrocyte numbers over the next 30-60 days [6]. Similar timescales have been observed for *Plasmodium vivax* infections [7].

Sensitivity

Whilst the WHO case definition of SMA does not include fever, it is plausible that children with acute malaria infections leading to severe anaemia are more likely to be febrile and subject to worse outcomes compared with those who happen to have both asymptomatic malaria infections and severe anaemia from another cause. To capture this modified syndromic representation of SMA, we adjusted the classification of MASA to further require the inclusion of (reported) fever in the 2 weeks prior to the survey in the definition. We assess the sensitivity of results to the diagnostic method used, by re-running the analysis using a malaria case definition ascertained by RDT. In the main analysis we adjusted for the rate of non-malarial severe anaemia (severe anaemia in the absence of a malaria infection). This definition is not the same as in the hospitalisation data (which does not adjust for non-malarial severe anaemia), although hospitalised severe anaemia may be more likely due to malaria than other less acute causes. We investigated the sensitivity to this assumption by refitting without the non-malarial severe anaemia adjustment. To avoid some parameters being unidentifiable, we specified a moderately informative prior on the duration of SMA in the main analysis, informed by published observations from humans and animal models. We tested the sensitivity of results to this specification by refitting with a much less informative prior.

Results were not as sensitive to the malaria diagnostic used or the lack of adjustment for non-malarial severe anaemia. Both sensitivity analyses would be expected to increase our estimates of the prevalence of malaria and severe anaemia leading to corresponding decreases in the estimate of the percentage of cases hospitalised. This occurred in all cases except the RDT-adjusted estimate for Kenya where the result is influenced by the estimate and associated adjustment for non-malarial anaemia not changing for microscopy and RDT.

Figures

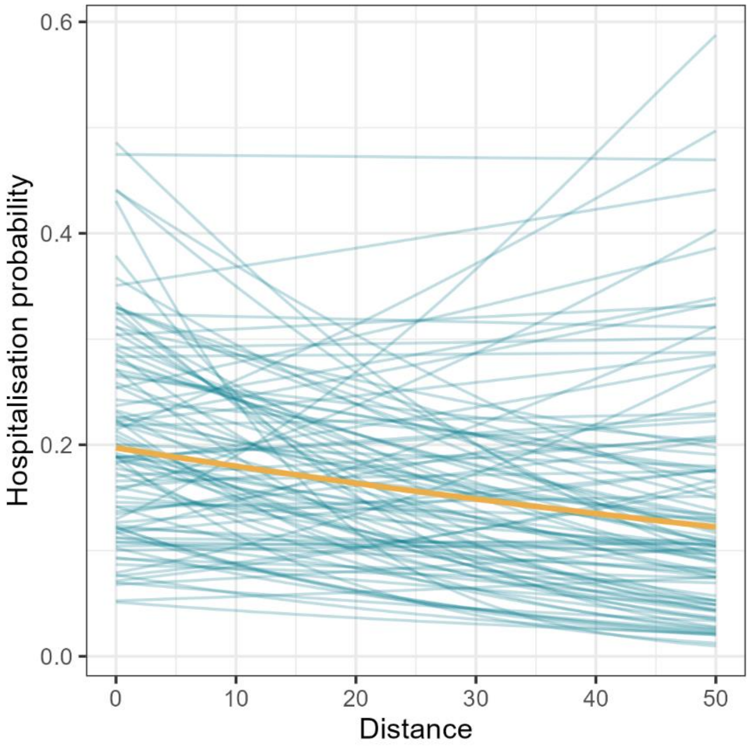

Figure S1. The relationship between hospitalisation probability and distance to hospital (km). We observed weak evidence for a decreasing hospitalisation probability as distance to the hospital increased.

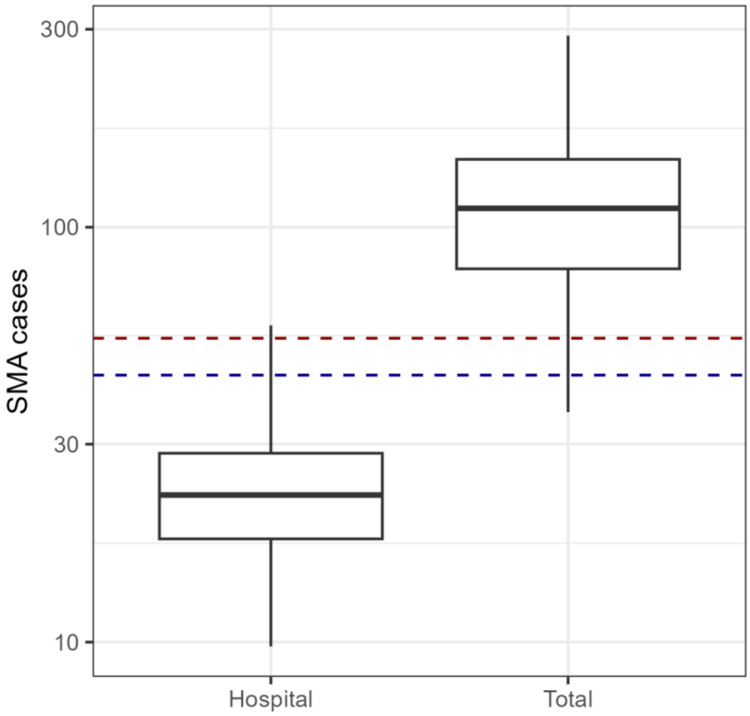

Figure S2. Validation of incidence model against RTS,S trial data. Boxplots show hospitalised and total combined incidence predictions (from 1,000 posterior samples) from the model given RTS,S trail reported $PfPr_{2-10}$. Dashed lines show trial reported SMA incidence given by two definitions: Plasmodium falciparum parasite density cut-off of >5000 per mm^3 (blue dashed line) and >0 per mm^3 (red dashed line) [2]. Box midline indicates the median, shoulders the 25% and 75% percentiles and whiskers the range from the analysis. All other things being equal we would expect the model prediction of total incidence to align with the observations (dashed lines). However, it is likely that other aspects relating to the monitoring, access to care, diagnosis and treatment of malaria in the trial population are different to the survey population.

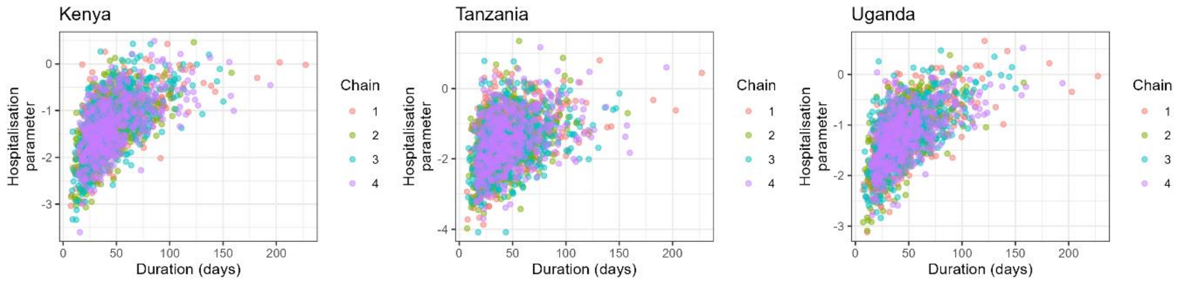

Figure S3. Correlation between duration of SMA and hospitalisation parameter, η. Points show 2,000 posterior draws from four chains for each of the three countries.

Table S1. Model parameters, definitions and prior specification.

Parameter	Definition	Prior	Notes	Ref
α	Intercept in the linear predictor for the upper asymptote of symptomatic SMA prevalence.	$Norm(0, 10)$	Noninformative	-
β	Country random effect in the linear predictor for the upper asymptote of symptomatic SMA prevalence.	$Norm(0, 10)$	Noninformative	-
γ	The shift of the symptomatic SMA prevalence curve (displacement with respect to $PfPr_{2-10}$)	$Norm(0, 10)$	Noninformative	-
δ	Growth rate of symptomatic SMA prevalence with respect to $PfPr_{2-10}$.	$Norm(0, 10)$	Noninformative	-
ζ	The recovery rate from SMA. Parameterised as ζ^{-1}, the average duration of symptomatic SMA.	$LogNorm(log(14), 0.7425)$	Weakly informative with a median of 14 days and upper 95% percentile of 60 days. Duration assumed to be greater than the average time until hospitalisation for SMA (approximately 7 days), and in line with rodent and monkey models and observations in humans. For sensitivity analysis, $sd = 1.66$, representing an upper 95% percentile of approximately 1 year. Parameters are on the log scale.	[4–9]
η	The intercept term for the linear predictor for the odds of hospitalisation of a SMA case.	$Norm(0, 1.5)$	Weakly informative. Set so the 95% percentiles for probability of hospitalisation are 5%, 95% to improve model convergence.	-
θ	Distance coefficient term in the linear predictor for the odds of hospitalisation of a SMA case.	$Norm(0, 10)$	Noninformative	-
σ	The standard deviation of the random effects.	$Unif(0,10000)$	Noninformative	-
μ	The overdispersion parameter of the negative binomial	$LogNorm(0,5)$	Non informative	-
$SA_{0.5-5i}$	Non-malarial severe anaemia prevalence	$Unif(0,1)$	Non informative	-

Table S2. Model parameters estimates. Estimates are summarised by the posterior median and 95% credible intervals (CrI).

		Posterior median estimate (95% CrI)				
**Parameter**	**Definition**	**Main analysis**	**Sensitivity: fever included in SMA definition**	**Sensitivity: RDT malaria diagnostic**	**Sensitivity: no adjustment for non-malarial severe anaemia**	**Sensitivity: uninformative prior on duration of SMA**
α	Intercept in the linear predictor for the upper asymptote of symptomatic SMA prevalence.	-5.7 (-6.1, -5.2)	-6.9 (-7.4, -6.4)	-5.4 (-5.8, -4.8)	-5.7 (-6.1, -4.9)	-5.7 (-6.1, -5)
γ	The shift of the symptomatic SMA prevalence curve (displacement with respect to $PfPr_{2-10}$)	1.3 (1.1, 1.5)	1.3 (1.1, 1.5)	1.3 (1.2, 1.5)	1.3 (1.1, 1.5)	1.3 (1.1, 1.5)
δ	Growth rate of symptomatic SMA prevalence with respect to $PfPr_{2-10}$.	6 (3.5, 9.1)	7.9 (5.5, 11)	6 (3.6, 8.8)	5.7 (2.7, 9.4)	5.8 (3, 9)
ζ	The recovery rate from SMA. Parameterised as ζ^{-1}, the average duration of symptomatic SMA.	41 (15, 100)	26 (9.5, 72)	54 (21, 140)	50 (18, 130)	74 (21, 230)
θ	Distance coefficient term in the linear predictor for the odds of hospitalisation of a SMA case.	-0.013 (-0.056, 0.031)	-0.02 (-0.066, 0.027)	-0.014 (-0.059, 0.029)	-0.01 (-0.054, 0.032)	-0.009 (-0.052, 0.034)
σ	The standard deviation of the random effects.	0.62 (0.41, 0.97)	0.77 (0.46, 1.3)	0.7 (0.47, 1.1)	0.63 (0.42, 1)	0.61 (0.41, 0.96)
μ	The overdispersion parameter of the negative binomial	3.4 (1.6, 6.9)	2.9 (1.4, 6.1)	3.3 (1.6, 6.9)	3.5 (1.7, 7.1)	3.4 (1.7, 7)

Table S3. Posterior estimates of the country hospitalisation and non-malarial severe anaemia parameters.

			Posterior median estimate (95% CrI)				
**Parameter**	**Definition**	**Country**	**Main analysis**	**Sensitivity: fever included in SMA definition**	**Sensitivity: RDT malaria diagnostic**	**Sensitivity: no adjustment for non-malarial severe anaemia**	**Sensitivity: uninformative prior on duration of SMA**
η	The intercept term for the linear predictor for the odds of hospitalisation of a SMA case.	Kenya	-1.3 (-2.6, -0.14)	-0.88 (-2.2, 0.42)	-1.1 (-2.3, 0.12)	-1.3 (-2.5, -0.16)	-0.85 (-2.2, 0.42)
		Tanzania	-1.5 (-3, 0.073)	-1.1 (-2.7, 0.54)	-2.5 (-3.8, -1.2)	-1.8 (-3.1, -0.44)	-1.1 (-2.7, 0.41)
		Uganda	-1.2 (-2.3, -0.096)	-1 (-2.2, 0.12)	-1.4 (-2.5, -0.32)	-1.4 (-2.5, -0.31)	-0.78 (-2, 0.36)
$SA_{0.5-5i}$	Non-malarial severe anaemia prevalence	Kenya	0.00058 (0.00029, 0.00099)	0.00047 (0.00024, 0.00081)	0.00059 (0.0003, 0.001)	-	0.00057 (0.00029, 0.00099)
		Tanzania	0.0024 (0.0015, 0.0036)	0.0023 (0.0014, 0.0035)	0.0017 (0.0009, 0.0028)	-	0.0024 (0.0015, 0.0036)
		Uganda	0.0011 (0.00056, 0.0018)	0.00095 (0.00051, 0.0016)	0.00079 (0.00034, 0.0015)	-	0.001 (0.00055, 0.0017)

Table S4. Posterior estimates of the country level random effect.

			Posterior median estimate (95% CrI)				
**Parameter**	**Definition**	**Country**	**Main analysis**	**Sensitivity: fever included in SMA definition**	**Sensitivity: RDT malaria diagnostic**	**Sensitivity: no adjustment for non-malarial severe anaemia**	**Sensitivity: uninformative prior on duration of SMA**
β	Country random effect in the linear predictor for the upper asymptote of symptomatic SMA prevalence.	Angola	-0.087 (-1.1, 0.82)	-0.32 (-2, 0.93)	0.21 (-0.77, 1.1)	-0.088 (-1.1, 0.85)	-0.084 (-1.1, 0.82)
		Benin	-0.51 (-1.1, 0.046)	-0.56 (-1.5, 0.25)	-0.74 (-1.4, -0.18)	-0.51 (-1.1, 0.057)	-0.53 (-1.2, 0.044)
		Burkina Faso	0.58 (0.15, 1)	-0.13 (-0.93, 0.61)	0.52 (0.08, 0.98)	0.6 (0.15, 1.1)	0.57 (0.13, 1.1)
		Burundi	0.85 (0.34, 1.4)	1.3 (0.63, 2)	1.1 (0.61, 1.6)	0.88 (0.36, 1.4)	0.85 (0.33, 1.4)
		DRC	0.3 (-0.27, 0.85)	0.89 (0.17, 1.6)	0.24 (-0.31, 0.78)	0.31 (-0.26, 0.87)	0.29 (-0.29, 0.85)
		Cote d'Ivoire	-0.79 (-1.8, -0.03)	-0.8 (-2.4, 0.25)	-0.81 (-1.7, -0.066)	-0.8 (-1.8, -0.043)	-0.8 (-1.8, -0.039)
		Ghana	0.028 (-0.58, 0.61)	-0.34 (-1.4, 0.55)	-0.088 (-0.71, 0.48)	0.046 (-0.58, 0.64)	0.023 (-0.59, 0.61)
		Guinea	0.19 (-0.46, 0.82)	0.29 (-0.66, 1.2)	0.42 (-0.18, 0.99)	0.19 (-0.49, 0.83)	0.18 (-0.48, 0.8)
		Kenya	-0.62 (-1.3, -0.018)	-0.29 (-0.99, 0.44)	-0.87 (-1.6, -0.23)	-0.68 (-1.4, -0.025)	-0.58 (-1.2, 0.035)
		Liberia	-0.6 (-1.6, 0.2)	-0.64 (-2.2, 0.45)	-0.85 (-2, -0.011)	-0.6 (-1.7, 0.21)	-0.6 (-1.6, 0.19)
		Madagascar	-0.31 (-1.2, 0.43)	-0.68 (-2.3, 0.41)	-0.48 (-1.4, 0.27)	-0.32 (-1.2, 0.44)	-0.32 (-1.2, 0.43)

		Malawi	0.58 (-0.023, 1.2)	1.1 (0.33, 1.9)	0.47 (-0.13, 1)	0.6 (-0.015, 1.2)	0.58 (-0.031, 1.2)
		Mali	1.2 (0.81, 1.6)	-0.47 (-1.3, 0.28)	0.96 (0.54, 1.4)	1.2 (0.77, 1.6)	1.2 (0.76, 1.6)
		Mozambique	0.017 (-0.63, 0.62)	-0.27 (-1.4, 0.64)	0.0038 (-0.62, 0.58)	0.021 (-0.63, 0.63)	0.0049 (-0.64, 0.62)
		Nigeria	0.071 (-0.42, 0.56)	-0.079 (-0.83, 0.65)	-0.09 (-0.59, 0.4)	0.089 (-0.41, 0.58)	0.066 (-0.42, 0.57)
		Rwanda	-0.17 (-1.3, 0.82)	0.14 (-1.3, 1.5)	-0.25 (-1.5, 0.79)	-0.16 (-1.3, 0.82)	-0.16 (-1.3, 0.83)
		Senegal	-0.16 (-0.99, 0.6)	0.37 (-0.65, 1.4)	0.07 (-0.71, 0.81)	-0.17 (-1, 0.61)	-0.17 (-1, 0.59)
		Sierra Leone	0.052 (-0.49, 0.58)	0.18 (-0.62, 0.96)	0.16 (-0.35, 0.67)	0.055 (-0.5, 0.6)	0.035 (-0.51, 0.58)
		Tanzania	0.15 (-0.55, 0.91)	0.61 (-0.058, 1.5)	0.84 (0.14, 1.5)	0.12 (-0.73, 0.91)	0.21 (-0.5, 0.95)
		Togo	-0.24 (-0.9, 0.37)	-0.0005 (-0.92, 0.85)	-0.38 (-1.1, 0.22)	-0.23 (-0.91, 0.39)	-0.25 (-0.92, 0.37)
		Uganda	-0.37 (-0.94, 0.18)	0.21 (-0.43, 0.88)	-0.39 (-0.99, 0.17)	-0.42 (-1.1, 0.17)	-0.31 (-0.89, 0.25)

Supplementary references

1. Roca-Feltrer A, Carneiro I, Smith L, Schellenberg JRA, Greenwood B, Schellenberg D. The age patterns of severe malaria syndromes in sub-Saharan Africa across a range of transmission intensities and seasonality settings. Malar J. 2010;9(1):1–9.

2. RTSS Clinical Trials Partnership. Efficacy and safety of RTS,S/AS01 malaria vaccine with or without a booster dose in infants and children in Africa: final results of a phase 3, individually randomised, controlled trial. Lancet [Internet]. 2015 Apr [cited 2015 Apr 26];386(9988):31–45. Available from: http://www.thelancet.com/article/S0140673615607218/fulltext

3. Agnandji ST, Lell B, Fernandes JF, Abossolo BP, Kabwende AL, Adegnika AA, et al. Efficacy and Safety of the RTS,S/AS01 Malaria Vaccine during 18 Months after Vaccination: A Phase 3 Randomized, Controlled Trial in Children and Young Infants at 11 African Sites. PLoS Med. 2014;11(7).

4. Evans KJ, Hansen DS, Rooijen N Van, Buckingham LA, Schofield L. Severe malarial anemia of low parasite burden in rodent models results from accelerated clearance of uninfected erythrocytes. 2006;107(3):1192–9.

5. Egan AF, Fabucci ME, Saul A, Kaslow DC, Miller LH. Brief report Aotus New World monkeys : model for studying malaria-induced anemia. 2002;99(10):3863–6.

6. Jakeman GN, Saul A, Hogarth WL, W CW. Anaemia of acute malaria infections in non-immune patients primarily results from destruction of uninfected erythrocytes. Parasitology. 1999;119:127–33.

7. Collins WE, Jeffery GM, Roberts JM. A retrospective examination of anemia during infection of humans with plasmodium vivax. Am J Trop Med Hyg. 2003;68(4):410–2.

8. Mousa A, Al-Taiar A, Anstey N, Badaut C, Barber B, Bassat Q, et al. The impact of delayed treatment of uncomplicated P. falciparum malaria on progression to severe malaria: a systematic review and a pooled multicentre individual-patient meta-analysis. PLoS Med. 2020;

9. Kurtzhals JAL, Adabayeri V, Goka BQ, Akanmori BD, Oliver-commey JO, Nkrumah FK, et al. Low plasma concentrations of interleukin 10 in severe malarial anaemia compared with cerebral and uncomplicated malaria. 1998;351.
